# Microglial Response to *Aspergillus flavus* and *Candida albicans*: Implications in Endophthalmitis

**DOI:** 10.3390/jof6030162

**Published:** 2020-09-05

**Authors:** Jaishree Gandhi, Poonam Naik, Inderjeet Kaur, Ashok Kumar, Joveeta Joseph

**Affiliations:** 1 Jhaveri Microbiology Centre, Brien Holden Eye Research Centre, L. V. Prasad Eye Institute, Hyderabad, Telangana 500034, India; jaishreegandhi123@gmail.com (J.G.); naikpoonam92@gmail.com (P.N.); 2 Manipal Academy of Higher Education, Manipal, Karnataka 576104, India; 3 Kallam Anji Reddy Molecular Genetics Laboratory, Jhaveri Microbiology Centre, Brien Holden Eye Research Centre, L. V. Prasad Eye Institute, Hyderabad, Telangana 500034, India; inderjeet@lvpei.org; 4 Department of Ophthalmology, Visual and Anatomical Sciences, Wayne State University, Detroit, MI 48202, USA; akuma@med.wayne.edu

**Keywords:** microglia, *Aspergillus* sp., *Candida* sp., endophthalmitis, innate immune

## Abstract

*Aspergillus flavus* is the most common etiology of fungal endophthalmitis in India, while *Candida albicans* is the causative agent in the West. In this study, we determined the role of microglial cells in evoking an inflammatory response following an infection with *A. flavus* and *C. albicans* strains isolated from patients with endophthalmitis. Microglia (CHME-3) cells were infected with *A. flavus* and *C. albicans* and the expression of Toll-Like Receptors (TLRs), cytokines and Matrix metalloproteinases (MMPs) were assessed at various time intervals. *A. flavus* infected cells induced higher expressions of TLR-1, -2, -5, -6, -7 and -9 and cytokines such as IL-1α, IL-6, IL-8, IL-10 and IL-17. In contrast, *C. albicans* infected microglia induced only TLR-2 along with the downregulation of IL-10 and IL-17. The expression of MMP-9 (Matrix metalloproteinase-9) was however upregulated in both *A. flavus* and *C. albicans* infected microglia. These results indicate that microglial cells have the ability to incite an innate response towards endophthalmitis causing fungal pathogens via TLRs and inflammatory mediators. Moreover, our study highlights the differential responses of microglia towards yeast vs. filamentous fungi.

## 1. Introduction

Fungal endophthalmitis is a rare clinical entity globally, however, in tropical countries such as India and China, this incidence varies from 17–30% of the total cases seen and represents a significant problem, associated with a delayed diagnosis and poor prognosis [1]. The spectrum of fungal agents causing endophthalmitis depends on the clinical setting and the geographical location. While *Candida* sp. (yeast) is the most frequent pathogen reported from the Western countries such as the USA and Europe, in tropical countries such as India, the etiology of fungal pathogens includes *Aspergillus* sp. mainly along with *Alternaria*, *Fusarium*, and *Curvularia* species [1,2,3,4]. Fungal endophthalmitis due to yeast (*Candida albicans*) are usually endogenous and differ from filamentous fungi (*Aspergillus* or *Fusarium* sp.) as treatment is often successful, whereas mold endophthalmitis is usually exogenous and often results in permanent loss of vision. The major mechanism responsible for increased virulence in clinical *Candida* isolates in addition to viral coinfections is also the overexpression of plasma membrane efflux pumps and the use of P-glycoprotein pump inhibitor associated drugs for the enhancement of antifungal activity is now being proposed [5]. A previous study in our laboratory showed a high rate of detection of fungal pathogens in nearly 50% of our culture-negative samples by a Next Generation Sequencing (NGS) analysis and was identified to be predominantly *Aspergillus* sp. [3], which suggests that fungal endophthalmitis might be more prevalent than previously believed, especially in the developing countries. In series from India [4], the outcome was unfavorable in more than 50% of the patients with fungal endophthalmitis despite providing intensive therapy and the administration of intravitreal steroids to promote a faster clearance of inflammation remains controversial [6]. The pathogenesis of fungal endophthalmitis involves complex host pathogen interactions resulting in severe intraocular inflammation, and retinal tissue damage. The immune response is critical to the survival of all pathogenic organisms, and along with phagocyting the pathogen, it also facilitates the repair and regeneration of inflamed tissue [7]. Possible virulence factors in fungal endophthalmitis depend largely on the virulence of the organism. Although there are similar characteristics between various fungi and their immune response, each fungus has varying virulence mechanisms resulting in differential immune responses [8]. Therefore, the personalized manipulation of the inflammatory response could offer strategies to control or prevent exacerbations in those diseases.

Microglia, which represents the resident mononuclear phagocyte population present in the central nervous system (CNS), are among the potential retinal glial cells involved in antimicrobial defense [9] that respond to local injury and infectious agents. Under pathological conditions, microglia along with macrophages release inflammatory mediators that control the neuroinflammatory response via an enhanced recruitment of peripheral tissue dendritic cells, neutrophils and lymphocytes [10]. The role of microglia along with Muller glial cells in the initiation of the innate response to live pathogens during endophthalmitis has already been reported earlier, [9,11], however, despite the increasing prevalence of fungal identification in the intraocular fluids from endophthalmitis patients, the effect of fungal infection on the inflammatory response in microglia is poorly defined. Understanding the available information on how microglial cells react and influence disease progression in fungal infections is important and warrants further investigation to mitigate the number of clinical cases of patients with fungal endophthalmitis not responsive to standard therapy [12]. Additionally, a previous 25 year study at our institute found the filamentous fungus *Aspergillus flavus* (*A. flavus*) as one of the most clinically relevant fungal pathogens [13] which is a less studied organism compared to its related and well known human pathogen, *A. fumigatus* [14,15,16]. The purpose of the present study was to study the hypothesis that microglia cells would have a differential inflammatory response to infection by *C. albicans* and *A. flavus* isolates.

## 2. Materials and Methods

### 2.1. Ethics Statement

All procedures were approved by the Institutional Review Board of the L V Prasad Eye Institute, dated 11th September 2018 (LEC 09-18-124).

### 2.2. Fungal Isolates

The fungal isolates used in the study were clinical strains of *C. albicans* (L-614/2017) and *A. flavus* (L-416/2018) isolated from patients diagnosed clinically with postoperative fungal endophthalmitis and were operated and treated at our institute, which is a tertiary referral center in South India. The demographic details of these patients are given in Table 1. Following a detailed biomicroscopic examination including a retinal examination, these underwent pars plana vitrectomy and the vitreous sample was collected and transported to the microbiology laboratory immediately, where it was cultured on appropriate bacterial and fungal media. All media were incubated at 37 °C while the fungal media were incubated at 25 °C for 2 weeks. While *Candida albicans* was identified by ViTEK 2, *Aspergillus flavus* was identified based on their colony characteristics and microscopic features of its conidia. The *A. flavus* isolate was stocked and preserved in water at room temperature, while the *C. albicans* isolate was preserved on Sabouraud dextrose agar slopes at room temperature.

### 2.3. Generation of Fungal Preparations

*C. albicans* was cultured in Sabouraud Dextrose broth at 150 rpm under 37 °C for 24 h. The cells were then counted in a haemocytometer after isolating them by centrifugation at 4 °C for 3000× *g* for 15 min. Similarly, *A. flavus* was cultured on SDA and conidia were harvested after 5 days at 37 °C by scraping into phosphate-buffered saline (PBS, Sigma, St. Louis, MO, USA). The mix was then filtered using a muslin cloth to avoid hyphal contamination and washed in PBS at 8000 rpm for 10 min. The pellet was then resuspended in PBS for enumeration as described earlier.

### 2.4. In Vitro Culture of Microglia Cells (CHME-3) and Fungal Cocultivation

Human microglial cells (CHME-3) [17] were maintained and serially passaged in a cellular grade culture flask (Nunc EasYFlask 75 cm^2^) in DMEM (Gibco™, Waltham, MA, USA) with 10% fetal bovine serum (FBS, Gibco™) and 1% antibiotic cocktail and maintained at 37 °C and 5% carbon dioxide in a humidified incubator. CHME-3 cells were grown to a confluence of 1.2 × 10^6^ cells mL^−1^ in 6-well plates in antibiotics and serum-free DMEM for 16 to 18 h prior to fungal stimulation. The cells were then cocultured with *Candida albicans* or *Aspergillus flavus* strains with an MOI of 5:1 at 37 °C in 5% CO_2_. The uninfected CHME-3 cells were cultured under the same medium conditions. Upon the completion of incubation, at various time points (6–24 h), the culture supernatant were assessed for growth and viability as described previously [18]. The culture supernatants were filtered using a 0.33 μm membrane filter to remove conidia and debris. The samples (both supernatants and cells) were stored at −80 °C prior to RNA extraction and the Enzyme-Linked Immunosorbent Assay (ELISA).

### 2.5. Total RNA Extraction and Quantitative Real-Time Polymerase Chain Reaction

Total RNA was extracted from the CHME-3 cells using the Qiagen RNeasy Mini kits and cDNA was synthesized using 1 µg of total RNA by Maxima first-strand cDNA synthesis kit (SuperScript; Verso). The cDNA was then amplified using gene-specific PCR primers for human Toll-Like Receptors (TLRs) (TLR1–TLR7 and TLR9), cytokines/chemokine (IL-1α, IL-1β, IL-6, IL-8, TNF-α, IL-10, IL-17, M-CSF), Matrix Metalloproteinases (MMPs) (MMP-2, MMP-9) and TIMP-1 (Tissue Inhibitor of Metalloproteinases) using SYBR Green (DyNAmo Flash SYBR Green qPCR Kit, Thermo Scientific, Waltham, MA, USA) on the Real-Time PCR system (Applied Biosystems Qauntstudio3 system). The real-time primers are listed in Supplementary Table S1. The quantification of gene expression was determined via the comparative ΔΔCT method. Gene expression in the test samples was normalized to the endogenous control β-actin, and was reported as the fold change relative to uninfected controls.

### 2.6. Enzyme-Linked Immunosorbent Assay (ELISA)

Interleukin-6 (sensitivity < 0.11 pg/mL), IL-8 (sensitivity < 0.13 pg/mL), IL-10 (sensitivity < 0.56 pg/mL), and GM-CSF (sensitivity < 0.35 pg/mL) concentrations in cell culture supernatant were determined using the MAGPIX Milliplex kit (Merck) following the manufacturer’s protocol. Untreated cells were taken as a negative control. Additionally, the MMP-9 concentration in culture supernatants was also measured using a sandwich ELISA (Sigma Aldrich, Merk) and read using a BT 2000 Microkinetics Reader at 450 nm. A standard curve was constructed to quantify the MMP-9 with a detection range of 8–6000 pg/mL.

### 2.7. Immunofluorescence Assay

For immunocytochemistry staining, microglia cells were grown on coverslips and infected with *A. flavus* and *C. albicans* for the indicated time period. The cells were washed three times with phosphate-buffered saline (PBS) and fixed with 4% paraformaldehyde and blocked in 2.5% BSA for 1 h at room temperature followed by an incubation with IL-17, and IL-1β (Abcam, Cambridge, UK) (1:200 dilution) antibodies overnight at 4 °C. Following the removal of the primary antibodies, the cells were then extensively washed with PBS and incubated with Invitrogen Alexa Fluor 594 goat anti-rabbit IgG secondary antibody prepared in 1% BSA in PBS at a dilution of 1:400 for 1 h at room temperature and mounted using Fluoroshield™ medium and visualized under a fluorescence microscope (Carl Ziess AXIO 503 monoscope A1). The level of fluorescence intensity were quantified using Image J version 1.44 software (National Institutes of Health, USA). The corrected total cell fluorescence was calculated from the regions of interest (ROIs) selected and the mean fluorescence values were plotted after subtracting the background value [19].

### 2.8. Heatmap and Network Analysis for Differentially Expressed Genes (DEGs)

A gene expression heatmap was constructed employing Differentially Expressed Genes (DEGs) in human microglial cells challenged with *A. flavus* and *C. albicans* at the desired time point. DEGs with a >2-fold change in at least three data sets were selected and a clustered heatmap was plotted using the online tool heatmapper.ca [20]. The heatmap colors displayed a normalized expression value (zero meant normalization of log2 transformed expression). Genes with significant differential expression were used to generated protein–protein interaction network (PPI) using STRING (Search Tool for the Retrieval of Interacting Genes/Proteins 9.1) [21] (http://string-db.org/) database. The results of the analyses generated a gene–protein interaction network where the intensity of the edges reflected the strength of the interaction score.

### 2.9. Statistical Analysis

All the values were presented as the mean ± SE. The statistical analysis was performed using Student’s *t*-test (GraphPad Prism software version 5.0) for a comparison of two groups. The following symbols were used to indicate statistical significance: * *p* ≤ 0.05 and ** *p* ≤ 0.005. All experiments were performed at least three times unless indicated otherwise.

## 3. Results

### 3.1. Toll-Like Receptors’ (TLRs) Expression Profile in Response to Aspergillus flavus and Candida albicans in Microglia

**Figure 1:**
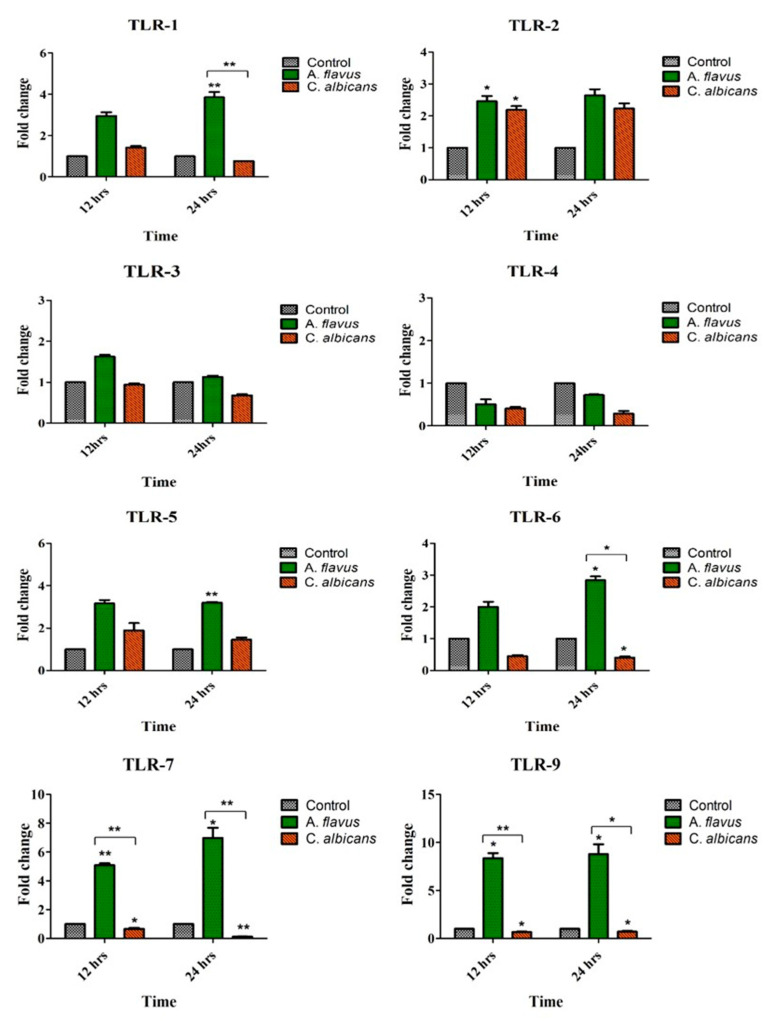
CHME-3 cells were challenged with *A. flavus* and *C. albicans* at indicated time points. Postinfection human microglial total RNA was subjected to RT-PCR and qRT-PCR using SYBR green assay for Toll-Like Receptors (TLRs) (1-7 and -9) and β-actin gene was used as internal control and uninfected CHME-3 cells were taken as control. The data were shown as the mean ± Standard error (SE) from three sets of independent experiments and * *p* < 0.05, ** *p* < 0.005.

A. flavus challenged microglial cells demonstrated an increased expression of various TLRs compared to uninfected cells, which included TLR-1 (3.8-fold; *p* < 0.005), TLR-5 (3-fold; *p* < 0.005) and TLR-6 (2.8-fold; *p* < 0.05) at 24 h postinfection (p.i.). Additionally, the mRNA expression of TLR-7 (5-fold; *p* < 0.005) and TLR-9 (8-fold; *p* < 0.05) were overexpressed at 12 h itself, while TLR-2 was slightly upregulated (2.4-fold) by microglia cells infected with *A. flavus* at the same time point (Figure 1).

In comparison, *C. albicans* challenged microglial cells demonstrated an increased expression of only TLR-2 (2.2-fold) at 24 h in comparison to uninfected microglia cells (Figure 1). TLR-3 and TLR-4, however, did not show any expression in cells challenged with both the fungal pathogens. To understand if human microglial cells have a differential response to conidia (*A. flavus*) and yeast-like fungus (*C. albicans*), we found that TLR-1, TLR-6, TLR-7 and TLR-9 were highly expressed in cells infected with *A. flavus* but downregulated in microglial cells challenged with *C. albicans* (Figure 1).

### 3.2. Induction of Immune Mediators by Microglia Challenged with A. flavus and C. albicans

**Figure 2:**
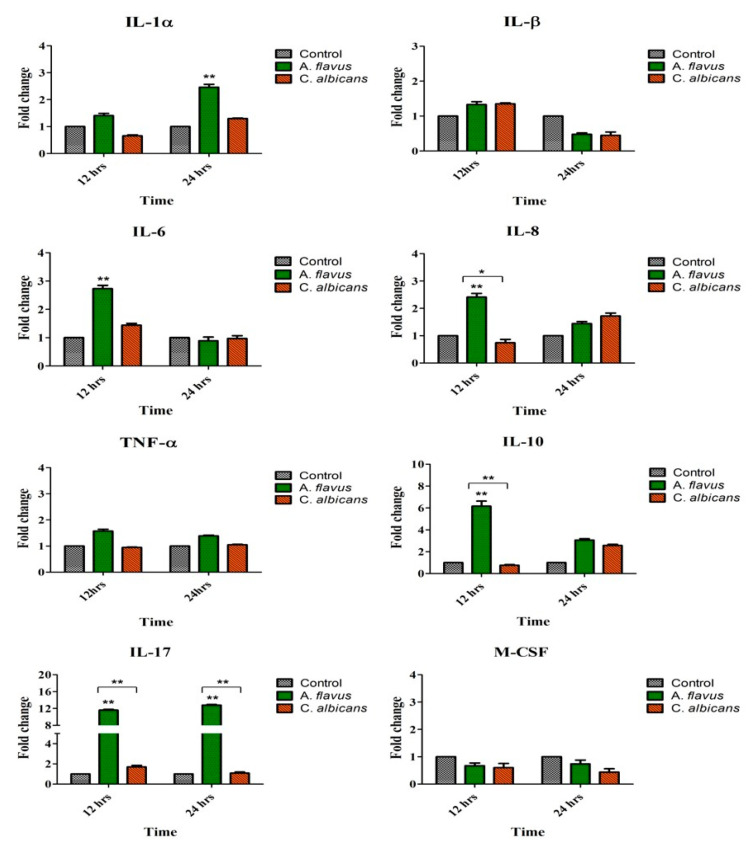
CHME-3 cells were challenged with *A. flavus* and *C. albicans* at indicated time points. Postinfection human microglial total RNA was subjected to RT-PCR and qRT-PCR using SYBR green assay with cytokine-specific primes. Representative RT-PCRs of each cytokines were shown. Human microglia with no fungal infection cDNA was used as control. β-actin was used as an endogenous control. Data are from n = 3 independent experiments done in triplicate. * *p* < 0.05, ** *p*< 0.005 compared to control.

Similar to TLRs’ activation of microglial cells by *A. flavus*, there was an increased expression of proinflammatory mediators such as IL-6 (2.7-fold) and IL-8 (2.4-fold) at 12 h postinfection while IL-1α (2.4-fold) was expressed at 24 h p.i. compared to uninfected cells. Similarly anti-inflammatory cytokine IL-10 (6-fold) was also upregulated at 12 h p.i. (*p* < 0.005) along with IL-17 (11.6-fold; *p* < 0.005) which showed continued increased expression up to 24 h p.i. (12.7-fold, *p* < 0.005) (Figure 2). There was however, no significant expression of IL1β, TNF-α and M-CSF in microglia cells challenged with both *A. flavus* as well as *C. albicans*. Interestingly none of the immune mediators tested were expressed in cells infected with *C. albicans*. Comparing the expression between the two fungal pathogens, we found that microglial cells infected with *A. flavus* were significantly upregulated in IL-8 (*p* < 0.05), IL-10 (*p* < 0.005) and IL-17 (*p* < 0.005) at 12 h p.i. whereas in *C. albicans* infected cells these mediators were downregulated at the indicated time points (Figure 2).

### 3.3. Expression of Tissue Remodeling Enzymes by A. flavus and C. albicans Infected Microglia

**Figure 3:**
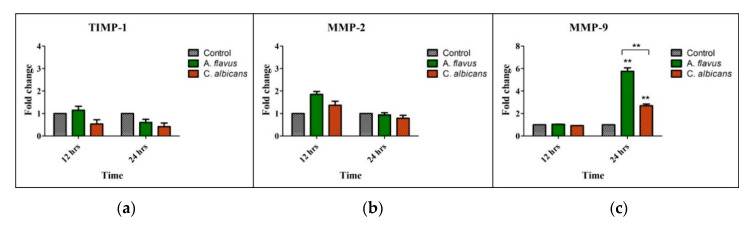
CHME-3 cells were challenged with *A. flavus* and *C. albicans* at indicated time points. The bar graphs show that there is no significant expression of (**a**) Tissue Inhibitor of Metalloproteinase-1 (TIMP-1) and (**b**) Matrix metalloproteinase-2 (MMP-2) in CHME-3 cells infected with *A. flavus* and *C. albicans*. (**c**) Significantly elevated expression of MMP-9 was observed in CHME-3 cells challenged with *A. flavus* and *C. albicans* compared with control. Data shown are the mean standard errors of three independent experiments performed in triplicate ** *p* < 0.005.

Matrix metalloproteinases’ (MMPs), such as MMP-2, MMP-9 and Tissue Inhibitor of metalloproteinases (TIMP-1), mRNA levels were assessed in CHME-3 cells challenged with *A. flavus* and *C. albicans* compared to uninfected cells and we observed an increased mRNA expression of only MMP-9 at 24 h p.i. (5.8-fold and 2.7-fold, respectively) (*p* < 0.005) (Figure 3c). However, the expression of MMP-9 in *A. flavus* infected cells was significantly higher (*p* < 0.005) than cells infected with *C. albicans* (Figure 3c). In comparison, MMP-2 and its inhibitor TIMP-1 had no significant expression in cells infected with both *A. flavus* and *C. albicans* (Figure 3a,b).

### 3.4. Protein Expression of Immune Mediators in Microglia Infected with Fungus

**Figure 4:**
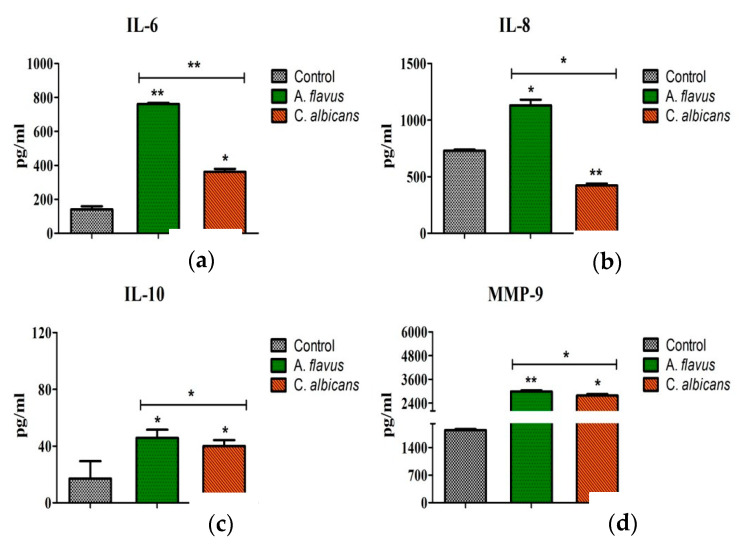
CHME-3 cells were infected with *A. flavus* and *C. albicans*. Production of (**a**) IL-6, (**b**) IL-8, (**c**) IL-10 and (**d**) MMP-9 were measured in cell-free supernatants after 24 h at 37 °C by ELISA. Values shown are the mean ± SE of three independent experiments performed in triplicate. Significant variations in production of cytokines and MMPs were determined by Student’s *t*-test. * *p* < 0.05, ** *p* < 0.005.

The translation of these mRNA transcripts was confirmed by assessing the protein levels of these immune mediators. Consistent with qRT-PCR data, significantly increased concentrations of IL-10 (46 pg/mL; *p* < 0.05), IL-6 (762 pg/mL; *p* < 0.005), and IL-8 (1130 pg/mL; *p* < 0.05) accumulated in the culture media of *A. flavus* stimulated CHME-3 cells at 24 h p.i. compared to *C. albicans* (IL-6 = 362 pg/mL; *p* < 0.05, IL-8 = 423 pg/mL; *p* < 0.005, IL-10 = 40 pg/mL; *p* < 0.05) (Figure 4a–c). The induction of MMP-9 levels in particular showed elevated levels in cells infected with both *A. flavus* and *C. albicans* (2993.66 ± 49.01 pg/mL vs. 2779.00 ± 74.22 pg/mL, *p* = 0.05) at 24 h postinfection. The protein concentrations of MMP-2 and MMP-9 were also assessed and MMP-2 was not detectable in the supernatant of both *A. flavus* and *C. albicans* challenged cells. MMP-9 however, showed a high expression in CHME-3 cells infected with *A. flavus* (2994 pg/mL ± 49; *p* < 0.005) compared to *C. albicans* (2779 pg/mL ± 74; *p* < 0.05) (Figure 4d).

**Figure 5:**
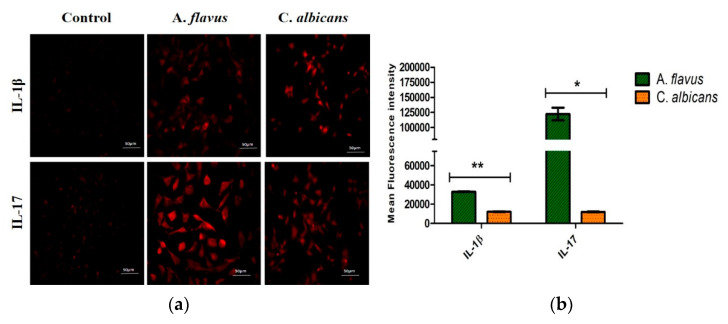
(**a**) Representative images of microglial cells infected with *A. flavus* and *C. albicans* were stained with anti-IL-17 and anti-IL-1β, labeled with Alexa 594 (red) after 12 h p.i. and uninfected CHME-3 cells were taken as control. Magnification 20×. (**b**) The bar graph depicting cell fluorescence intensity (normalized to background) in microglial cells challenged with *A. flavus* and *C. albicans* measured using NIH Image J software. Relative immunofluorescence staining was quantified from randomly selected fields. Values represent the mean fluorescence intensity ± SE (gray-value of 16-bit images). * *p* < 0.05, ** *p* < 0.005.

To confirm the mRNA expression observed for the remaining mediators, immunofluorescence staining was also carried out for the IL-17 and IL-1β expression in CHME-3 cells challenged with both *A. flavus* and *C. albicans* (Figure 5a), and the fluorescence intensities were compared to the uninfected cells at 12 h p.i. Similar to mRNA transcription levels, a higher protein expression of IL-17 was observed in cells infected with *A. flavus* compared to those infected with *C. albicans*. IL-1β however, showed a moderate expression in CHME-3 cells challenged with both *A. flavus* and *C. albicans* (Figure 5b).

### 3.5. Heatmap and PPI Network

**Figure 6:**
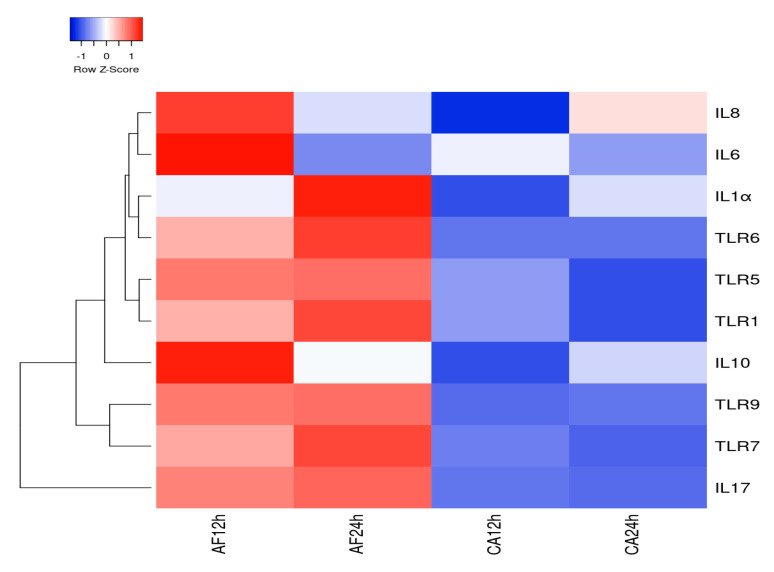
Gene expression heatmap constructed employing heatmapper.ca for 10 differentially expressed genes in human microglial cells challenged with *A. flavus* (AF) and *C. albicans* (CA) at 12 and 24 h p.i. The cluster value is coded with a range of colors from blue (for the lowest expression level) to red (for the highest expression level).

The heatmap analysis for 10 DEGs by human microglial cell infected with *A. flavus* and *C. albicans* demonstrates that the maximum gene expression was at 12 and 24 h by microglial cells challenged with *A. flavus*. Specifically, genes such as IL-6, IL-8 and IL-10 showed a high expression at 12 h; TLR-1, TLR-6 and IL-1α exhibited a high expression at 24 h in comparison to the other genes included in the analysis (Figure 6). However, the gene expression heatmap for *C. albicans* infected microglial cells revealed a lower expression level even at a higher MOI (10:1) in comparison to genes expressed by microglial cells infected with *A. flavus* except for IL-8 (Figure 6). Thus, there is a clear distinction in terms of the gene expression levels in microglial cells challenged with *A. flavus* vs. *C. albicans*. The hierarchical cluster analysis of real-time data was obtained using Heatmapper (http://www2.heatmapper.ca/) demonstrates that DEGs (differentially expressed genes) precisely distinguish human microglial cells (CHME-3) challenged with *A. flavus* from *C. albicans*.

**Figure 7:**
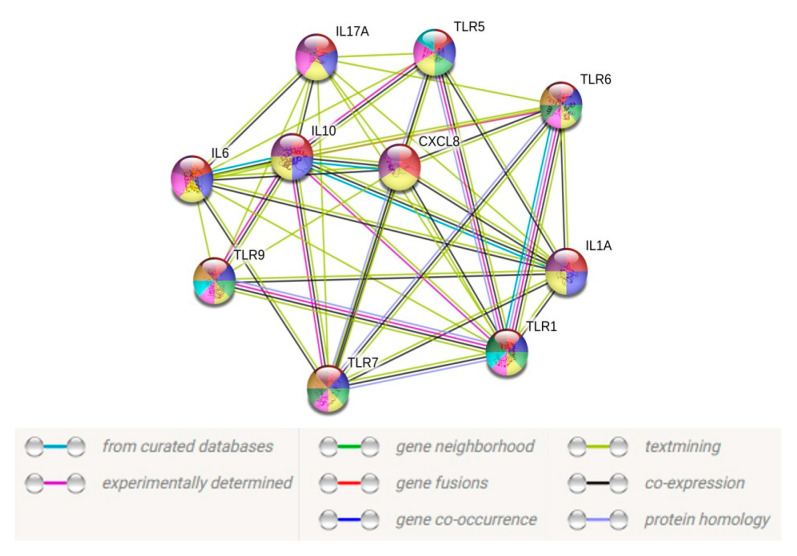
Protein–protein interaction network for 10 target genes with a medium confidence level 0.40. Connecting line color indicates the type of information used to infer the interaction. Purple line indicates experimental evidence; blue line indicates database evidence; green line indicates gene neighborhood; red line indicates gene fusion; dark blue indicates gene co-occurrence; yellow line indicates text mining; black line indicates coexpression of genes; light blue indicates protein homology.

To unveil the functional facets associated with these TLRs and cytokines, we constructed a protein–protein interaction (PPI) network using the STRING database (https://string-db.org/) based on the transcript data obtained from the CHME-3 infection with *A. flavus* and *C. albicans*. Only 10 TLRs and/or cytokines with a fold change of >2-fold were utilized to construct the PPI network. By excluding the proteins without a quantified or differential expression, our resulting PPI network contained 10 nodes and 62 edges (Figure 7), with a *p*-value < 1.0 × 10^−16^, indicating a close interaction, while the PPI for CHME-3 infected with *Candida* sp. did not show any significant interaction due to less than five differentially expressed genes.

## 4. Discussion

Vision loss in fungal endophthalmitis is due to pathogen virulence factors and host-evoked inflammatory damage to the retina along with the timing and mode of interventions [22,23]. Moreover, the ocular toxicity associated with the current antifungal drugs [24], the emergence of drug resistance [25] and limited clinical experience, make the treatment of these infections challenging.

Restricted treatment options in turn alters patient management, and thus it is critical to develop better therapeutics, and to improve diagnostics and intervention strategies.

Over the last few decades, active research is being directed to understand the effect of fungal pathogens on cells as well as the host–pathogen interplay, including the secretion of cytokines and to study the cross-talk between the major signaling pathways [24,26]. We have previously demonstrated [9], using a C57BL/6 mouse model of bacterial endophthalmitis, that microglia plays an important role in initiating early innate responses to bacterial endophthalmitis and that both primary cells and the mouse-derived BV-2 microglia cell line respond the same way to infection. Continuing on from that report, we wanted to explore the responses to fungal strains isolated from patients with endophthalmitis in human microglial cell lines. Microglia are the resident mononuclear phagocytic cells and have been shown previously to play an important role in antifungal immunity in systemic fungal infections in mice by *C. albicans,* leading to retinal microglia activation, through the expression of TLRs [27]. However, Shah et al. [28] have reported that these microglia get activated due to the expression of dectin-1 via the recognition of fungal β-glucans, a major receptor present within fungal cell walls which in turn represses cytokine/chemokine production [29]. Hence, understanding the signaling pathways that govern the responses to both yeasts and molds in microglial cells is important. While *Candida* spp. remain the predominant cause of fungal endophthalmitis worldwide, in tropical countries such as India and China, *Aspergillus* spp. are the leading etiological agent of exogenous endophthalmitis [13]. However, the pathogenesis of *Aspergillus* endophthalmitis is not well studied, baring a report to evaluate the therapeutic efficacy of existing and newer antifungal agents in the eye of an animal model of *A. fumigatus* [24]. Keeping our focus on *A. flavus*, which has been described by us earlier as the most common etiology in our country [3,13], our study, for the first time, used microglia cells as a model for fungal endophthalmitis.

While the innate immune mechanisms of hosts infected with *Candida albicans* have already been studied, little is known about their role during endophthalmitis caused by filamentous fungi, *A. flavus.* In this study, we have shown that there would be a differential immune response to yeast and filamentous fungi by microglia cells. This difference in innate response among fungal pathogens is primarily speculated to involve cell wall components and their interaction with host cells and tissues, favoring fungal growth and allowing dissemination in the host [30]. The outer layer of the *C. albicans* cell wall is said to be packed with mannoproteins and cross-linked to β-1,6-glucans and, unlike *Aspergillus* spp., α-glucan is absent from *Candida* spp. cell walls [31]. *C. albicans* has also been hypothesized to initiate its own uptake by host cells via complement proteins or complement receptors as an alternative immune evasion strategy [32]. It has also been reported that these mannoproteins are glycosylphosphatidylinositol-modified during interactions with the host, allowing the activation of the immune response by masking the β-glucan layer and thereby leading to an ineffective activation of the host immune system [31]. Similarly, it has been reported that *C. albicans* tended to cause higher inflammatory cytokine and chemokine concentrations, along with increased neutrophils and inflammatory monocytes, compared with nonalbicans *Candida* infections [33]. Additionally, the MAPK signaling pathway was also demonstrated by Galan-Diéz et al. [30] to be involved in the process of β-glucan masking. Comparatively, the cell wall of *Aspergillus* sp. is a two-layered structure, consisting of a polymer of β-1,3-glucan, cross-linked to α-1,3-glucan, galactomannan, galactosaminogalactan and chitin, all of them covalently bound one to the other. Galactosaminogalactan, an adhesin that facilitates the binding of hyphae to macrophages, neutrophils, and platelets has been reported to be associated with masking cell wall β-glucans from recognition by dectin-1, decreased polymorphonuclear neutrophil apoptosis via an NK cell-dependent mechanism and ROS production. It is also required for stimulating IL-17 production by neutrophils as well as IL-1α, IL-12, and TNFα release as also seen in our study [32].

The presence of fungal-derived ligands following a direct recognition by Pattern Recognition Receptors (PRRs) through TLRs has also been reported, as has the involvement of TLR-2 and -4 in the mediation of host responses during systemic candidiasis as well as in corneal infections, which instigates the production of inflammatory cytokines [27,34,35]. Our results, however, show the expression of only TLR-2, on the surface of microglia by *C. albicans* which recognizes the carbohydrates such as mannose and β-glucans on the surface of *C. albicans* [34], while cells infected with *A. flavus* demonstrate then activation of several TLRs including TLR-1, -2, -5, -7 and TLR-9 have an important role in the initial interaction of this fungus with the host cells. While, TLR-9 is known to modulate the immune response towards conidia *A. fumigatus*, which in turn is specific for dectin-1 expression and the activation of IL-17 [36]. In addition, TLR-4 was also expressed by microglial cells infected with *A. flavus* at the 3 h time point (data not shown) but wasn’t expressed in cells infected with *C. albicans*. The initial expression of TLR-4 by *A. flavus* corroborates with reports that suggest only TLR-4-mediated proinflammatory signals (and not TLR-2 induced anti-inflammatory signals), are lost on *Aspergillus* germination to hyphae. Our findings also corroborated with studies showing initiation of an immune response via TLR signaling in the murine model of *Aspergillus fumigatus* endophthalmitis [37,38]. TLR-2 has been reported to be induced by unidentified ligands present on both hyphal and conidia forms of *A. fumigatus*, whereas ligands for only conidial forms are reported by TLR-4 [39,40]. Another study found the α-glucan of *Candida* sp. to play a major role in TLR2-mediated attenuation of the immune response, while TLR4-attenuation was caused by the presence of the α-, β-glucan, and galactomannan components of the *Aspergillus* cell wall [41]. Our findings are also in contrast to Koutsouras et al., who reported an induction of a proinflammatory response that is critical in the protection against fungi via TLR-4 signaling on the surface of microglial cells [10]. This could be due to the time points chosen in the study and perhaps an earlier time point could reveal the presence of TLR-4 activation. This indicates that TLRs discriminate between distinct fungal morphotypes [40].

In addition, *Aspergillus* hyphae, but not *Aspergillus* conidia, stimulated the production of IL-10 through TLR2-dependent mechanisms [40], as was also shown by the high expression of IL-10 by the 12 h time point. The conidia of *A. flavus* in turn lead to the activation of inflammatory responses by secreting proinflammatory cytokines, particularly, the cytokines IL-1α, IL-6, IL-8 and IL-17 as well as the anti-inflammatory cytokine IL-10. Comparatively, only IL-6 and IL-10 were expressed slightly in cells infected with *C. albicans* in our study. While there is limited knowledge on the modulation of anti-inflammatory processes by microglia in the setting of fungal infections, a previous study had shown that, an infection with *C. albicans* (in mice) induces immunosuppression by activating TLR-2, leading to the release of IL-10 [42]. Additionally, we noticed that *A. flavus* causes a downregulation in the levels of GM-CSF and IFN-γ in microglia cells, which was similar to the findings by Schneider et al. [43] who reported that *A. fumigatus* actively impairs the release of IFN-γ (and M-CSF) by NK cells. In contrast to the results obtained regarding the cytokine expression in the murine model of *Aspergillus* endophthalmitis [14], where the production of several inflammatory mediators including IL-1β, IL6, and CXCL2 were reported, we observed a nonsignificant downregulation of IFN-γ and IL-1β. Numerous observations that IL-17 contributes to fungal protection have been obtained from studies with experimental models of infection [44]. However, the exact role of IL-17 in controlling these fungi in humans is yet to be determined. Our study shows the overexpression of IL-17 in *A. flavus* infected cells but a downregulation in cells infected with *C. albicans*. Our observations of microglia cells’ impaired immune response when infected with *C. albicans* could be due to biofilm formation as described previously, which seems to protect *C. albicans* from microglial damage by impairment of fungal cell phagocytosis, the release of cytokines and chemokines, and NO production [42]. Although the phenotypic variations and activation of microglial cells are complicated, they are mainly said to be involved in anti-inflammatory and/or proinflammatory processes, depending on the infectious stimuli, and get polarized into multiple phenotypes or switch activity states [45]. Humans with a MyD88 signaling defect do not have a higher susceptibility to fungal infections [46]. However, TLR polymorphisms alters signaling pathways, thereby increasing the risk of fungal infections [47]. Some reports also suggests that *A. fumigatus* causes the activation of the TLR9/BTK/calcineurin/NFAT signaling pathway, causing the activation of several proinflammatory mediators, such as TNF-α, leading to early neutrophil recruitment, and fungal clearance [47]. A major limitation of our study is that it is an in vitro study with a human, immortalized CNS-derived microglial cell line, and while the characteristics of brain- and retinal-derived microglial cells are similar, the environments in which they reside as well as pathophysiological conditions might differ. It is clear that antigenic, structural, and transcriptional differences exist between cohorts of microglia [48]. IL-34 appears to be exclusive to retinal microglia and different levels of CD11c, CD11b, and TLR4, which have been shown in microglia cells of retinal origin. While Huang et al. reported that repopulated retinal microglia might share a similar origin as the brain [49], Hammond et al. compared the transcriptional data of retinal microglia to brain microglia and found a similar set of lineage-specific factors shared by both populations, suggesting that developing retina and brain microglia may be ontogenically similar [50]. Additionally, O’Koren et al. [51] have reported functional differences by niche and IL-34 dependency across two anatomically distinct locations in the retina during homeostasis. Consequently, their close communication with immune cells, in conjunction with their ability to tailor their function specifically to the surrounding milieu, we believe that differential inflammatory expression shown by our fungal isolates in brain microglial cells would be applicable in retinal microglial cells as well. Additionally, establishing pure primary retinal microglial cultures from human tissues is rather challenging and probably impossible, but considering the importance of Muller and microglial cell interactions in microglial cell signaling, it would also be of considerable importance to perform these studies with cocultures of microglial and Muller cells and future work could focus on this aspect.

## 5. Conclusions

In conclusion, we demonstrated that *A. flavus* infection initiates a host immune response by the activation of several TLRs, followed by an induction of inflammatory cytokines whereas *C. albicans* infection in microglia demonstrated an impaired level of proinflammatory cytokine release. Therefore, taking into account our results, we propose that microglia may be inefficient for the clearance of *C. albicans* from the retina during endophthalmitis. Conidia and yeasts have a differential immune response and understanding this and other mechanisms of inflammation will further our ability to initiate strategies of immunomodulation, to minimize the retinal damage associated with endophthalmitis. Thus, this study advances the role using immunomodulators such as natural extracts and essential oils [52,53,54] or phytochemicals in mitigating the impact of inflammatory molecules as some compounds have already been shown to be effective in the treatment of multidrug resistance infections [55]. Similarly, a commercial ophthalmic solution containing PVP-I 0.6% [56] and the more recently approved keratosept, containing hexamidine diisethionate 0.05%, used to aid in a faster re-epithelization, showed rapid antifungal activity against different *Candida* strains [57], and also represent promising compounds for adjuvant therapy. To the best of our knowledge, this is the first in vitro model of *A. flavus* induced infection and further detailed studies are required to understand the microglia interactions with the retina and thus contribute to better management of patients affected by *Aspergillus* endophthalmitis.

## Figures and Tables

**Table 1:** Clinical and Demographic details of the patients diagnosed clinically with fungal endophthalmitis.

Demographic Characteristics	*A. flavus*	*C. albicans*
Age	66 years	54 years
Gender	Female	Male
Initial Visual Acuity	HM+	20/80p
Final Visual Acuity	HM+	PL + PR Inaccurate
Microbiology	*Aspergillus flavus*	*Candida albicans*

## Supplementary Materials

The following are available online at https://www.mdpi.com/2309-608X/6/3/162/s1, Table S1: List of Human primers (TLRs, Cytokines/chemokine, MMPs and TIMP) used for qRT-PCR analysis.
