# Supplementary material for: Microglial Response to Aspergillus flavus and Candida albicans: Implications in Endophthalmitis
Source: J Fungi (Basel). 2020 Sep 5;6(3):162. doi: 10.3390/jof6030162 (PMC7558867; doi:10.3390/jof6030162)
Supplement: Supplementary file 1 [file jof-06-00162-s001.pdf]

Table S1. List of Human primers used for RT-qPCR analysis

S.No.	Gene	Primer Sequence	Product Size (bp)	Tm (^{o}C)
1	**TLR-1**	Fw: GAAGATTTCTTGCCACCCTAC<br>Rw: GAACACAATGTGCAGACTCTC	**271**	**62**
2	**TLR-2**	Fw: CTGGACAATGCCACATAC<br>Rw: CTAATGTAGGTGATCCTG	**210**	**56**
3	**TLR-3**	Fw: CACTATGCTCGATCTTTCCTAC<br>Rw: CAATTCAGGTACCTCACATTG	**159**	**62**
4	**TLR-4**	Fw: CAGAACTGCAGGTGCTGG<br>Rw: GTTCTCTAGAGATGCTAG	**197**	**56**
5	**TLR-5**	Fw: TTGCTCAAACACCTGGACAC<br>Rw: CTGCTCACAAGACAAACGAT	**149**	**60**
6	**TLR-6**	Fw: CTATTGTTAAAAGCTTCCATTTTGT<br>Rw: ACCTGAAGCTCAGCGATGTAGTTC	**187**	**60**
7	**TLR-7**	Fw: CTGACCACTGTCCCTGAG<br>Rw: AACCCACCAGACAAACCA	**264**	**62**
8	**TLR-9**	Fw: CCA CCC TGG AAG AGC TAA ACC<br>Rw: GCC GTC CAT GAA TAG GAA GC	**161**	**62**
9	**IL-1α**	Fw: CAGTTGCCCATCCAAACTTGT<br>Rw: ATAGAGGGTGGCCCCCC	**76**	**60**
10	**IL-1β**	Fw: CCTGTCCTGCGTGTTGAAAGA<br>Rw: GGGAACTGGGCAGACTCAAA	**149**	**60**
11	**IL-6**	Fw: GTAGCCGCCCCACACAGACAGCC<br>Rw: GCCATCTTTGGAAGGTTC	**174**	**60**
12	**IL-8**	Fw: TCTGCAGCTCTGTGTGAAGGT<br>Rw: TGAATTCTCAGCCCTCTTCAA	**252**	**60**
13	**IL-10**	Fw: GCTGGAGGACTTTAAGGGTTACCT<br>Rw: CTTGATGTCTGGGTCTTGGTTCT	**108**	**60**
14	**IL-17**	Fw: CATCCATAACCGGAATACCAATA<br>Rw: TAGTCCACGTTCCCATCAGC	**245**	**60**
15	**TNF-α**	Fw: CCCCAGGGACCTCTCTCTAATC<br>Rw: GGTTTGCTACAACATGGGCTACA	**94**	**60**
16	**M-CSF**	Fw: TTGGGAGTGGACACCTGCAGTCT<br>Rw: CCTTGGTGAAGCAGCTCTTCAGCC	**248**	**60**
17	**TIMP-1**	Fw: AGACCTACACTGTTGGCTGTGAG<br>Rw: GACTGGAAGCCCTTTTCAGAG	**130**	**56**
18	**MMP-2**	Fw: CAGGGAATGAGTACTGGGTCTATT<br>Rw: ACTCCAGTTAAAGGCAGCATCTAC	**119**	**60**
19	**MMP-9**	Fw: CACTGTCCACCCCTCAGAGC<br>Rw: GCCACTTGTCGGCGATAAGG	**263**	**56**
20	**β-actin**	Fw: ACTTAGTTGCGTTACACCCTT<br>Rw: GTCACCTTCACCGTTCCA	**156**	**56**
